# Supplementary material for: SARS-CoV-2 Neutralizing Antibodies in Three African Countries Following Multiple Distinct Immune Challenges
Source: Vaccines (Basel). 2024 Mar 27;12(4):363. doi: 10.3390/vaccines12040363 (PMC11054809; doi:10.3390/vaccines12040363)
Supplement: Supplementary file 1 [file vaccines-12-00363-s001.zip › vaccines-2908818-supplementary.pdf]

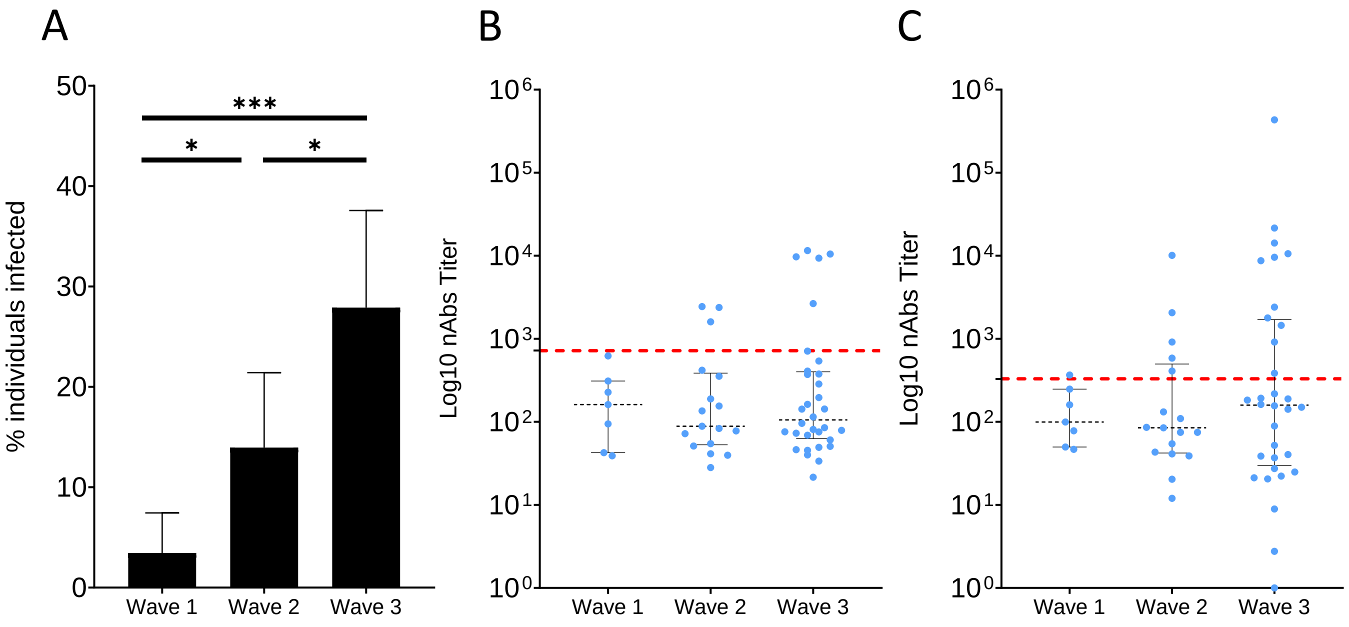


**Figure S1: nAb responses in I+V- HCWs in Madagascar according reported wave of infection.** (A) Percentage of unvaccinated HCWs who declared having been infected only once: during waves 1, 2 and 3. (B-C) SARS-CoV-2 specific nAb titers according to the declared wave of infection in individuals infected just once. (B) against W, (C) against BA.2. * *p-value* <0.05; *** *p-value* <0.0001, ns: non significative

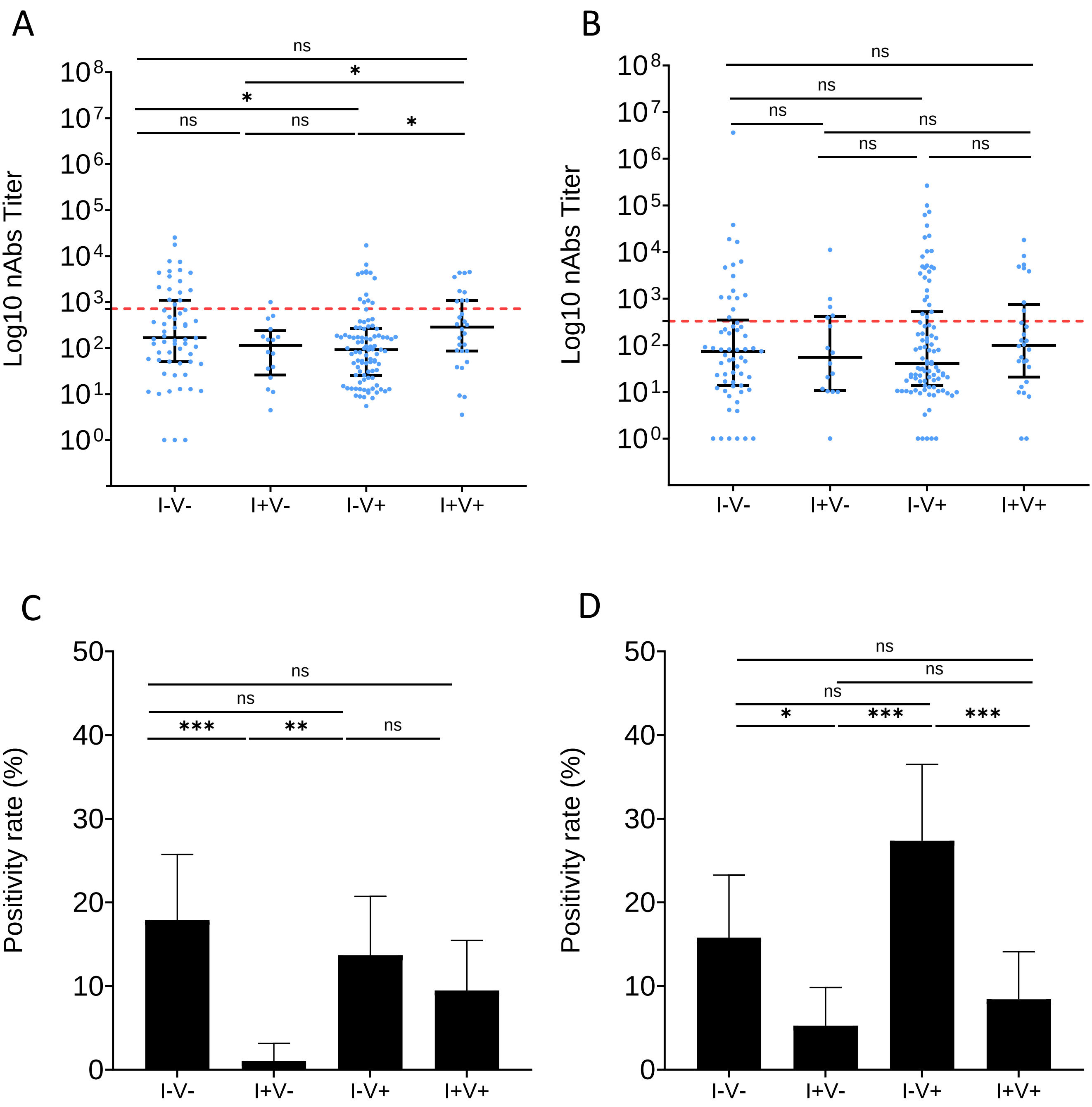


**Figure S2: Neutralizing antibody responses in the Cameroonian HCW sub-populations.** (A-B) SARS-CoV-2 specific nAb titers according to the type of immunization. (A) against WT, (B) against BA.2. populational groups are indicated on the X axis and neutralization titers on the Y axis. Data were compared using the Mann-Whitney test. Each dot represents an independent sample. (C-D) Percentage of individuals having nAb titers above the calculated threshold. (C) against WT, (D) against BA.2. Data were compared using the Fischer's exact test. * *p-value* <0.05 ; ** *p-value* <0.001; *** *p-value* <0.0001; ns: non significative

| Vaccines | | Madagascar n(%) | RCA n(%) | Cameroon n(%) |
|---|---|---|---|---|
| **ASTRAZENECA** | Dose1 | 131(39,82%) | 82 (70,69%) | 7 (5,69%) |
| | Dose2 | 117(35,56%) | 3 (50%) | |
| | Dose3 | 4(1,22%) | | |
| **Johnson&Johnson** | Dose1 | 176(53,5%) | 30 (25,86%) | 85 (69,10%) |
| | Dose2 | 7(2,13%) | 5 (83,33%) | 2 (1,63%) |
| | Dose3 | 2(0,61%) | | |
| **SINOPHARM** | Dose1 | 1(0,3%) | 2 (1,72%) | 29 (23,57%) |
| | Dose2 | 1(0,3%) | | |
| | Dose3 | | | |
| **Sinovac** | Dose1 | 1(0,3%) | | |
| | Dose2 | | | |
| | Dose3 | | | |
| **Moderna** | Dose1 | 1(0,3%) | | |
| | Dose2 | 2(0,61%) | | |
| | Dose3 | 1(0,3%) | | |
| **Pfizer/BioNTech** | Dose1 | 19(5,78%) | | |
| | Dose2 | 28(8,51%) | | |
| | Dose3 | 43(13,07%) | | |
| **Spoutnik V** | Dose1 | | | |
| | Dose2 | 1(0,3%) | | |
| | Dose3 | | | |
| **Unknow** | Dose1 | | 2 (1,72%) | 2 (1,62%) |
| | Dose2 | | 1 (16,66%) | |
| | Dose3 | | | |

**Table S1: .** Descriptive table of vaccines received during the study. The percentage corresponds to the number of individuals among the vaccinated individuals who received a type of vaccine (Dose 1: first vaccination, Dose 2: Second vaccination, Dose 3: Third vaccination).
